# Neonatal Kawasaki disease: Case report and literature review

**DOI:** 10.1097/MD.0000000000024624

**Published:** 2021-02-19

**Authors:** Cancan Li, Yiming Du, Huawei Wang, Gaohong Wu, Xueping Zhu

**Affiliations:** Department of Neonatology, Children's Hospital of Soochow University, China.

**Keywords:** diagnosis, kawasaki disease, neonate, newborn

## Abstract

**Rationale:**

Kawasaki Disease (KD) is a self-limiting and acute systemic vasculitis of childhood that leads to coronary artery abnormality in about 25% of untreated cases. KD is extremely rare in neonates. The purpose of this paper is to explore the clinical features and diagnosis and treatment of Neonatal Kawasaki Disease for early identification.

**Patient concerns:**

A 24-day-old male with 3 hours fever and a rash was admitted to our hospital.

**Diagnoses:**

He had a fever, rash, cracking of lips, lymph node enlargement in the neck, and distal extremity desquamation.

**Interventions:**

The patient was given intravenous immunoglobulin and aspirin with no complications.

**Outcomes:**

After discharge, the patient was followed up to 1 year old, with good prognosis and no carditis or coronary artery abnormalities.

**Lessons:**

Neonatal Kawasaki disease is extremely rare, and its clinical manifestation is not typical and easy to be missed. If not treated early, it will potentially give rise to coronary artery aneurysms or expansion, ischemic heart disease, and sudden death. Early diagnosis and treatment are very important.

## 1 Introduction

Kawasaki Disease (KD) is a self-limiting and acute systemic vasculitis disease of childhood that leads to coronary artery abnormality in about 20% to 25% of untreated cases. It is frequent in children under 5 years old. It is the leading cause of acquired heart disease in children in developed countries.^[1,2]^ KD commonly results in childhood heart disease, potentially giving rise to coronary artery aneurysms or expansion, ischemic heart disease, and sudden death.^[3]^ In some patients, heart disease might even affect the quality of their adult lives.^[4]^ Domestic and foreign reports of neonatal KD (NKD) are rare, and due to the lack of typical clinical manifestations, it is easy to be misdiagnosed, missed diagnosis and delayed treatment. We present a case of complete Kawasaki disease in a neonate of our hospital and a review of the literature on NKD. The aim is to explore the clinical features of NKD, to identify and diagnose it early, treat it as soon as possible, improve its prognosis and provide help for clinical practice.

## 2 Case presentation

A 24-day-old full-term male, admitted to our hospital, due to “fever for 3 hours with a rash”. The child developed fever without obvious inducement 3 hours earlier, with a body temperature of 38.5°C, foaming, sneezing, decreased intake of milk, and irritability.

A physical examination on admission, with a body temperature of 37.7°C, a pulse rate of 138 beats/min, and a respiratory rate of 44 beats/min. Polymorphous erythema was noted over the boy's facial ministry. His lung and heart were unremarkable at the examination. The umbilicus was clean and dry, and his extremities were felt warm.

Laboratory investigations included white blood cells (13.6 × 10^9^/L, neutrophils 56%), hemoglobin (132 g/L), platelets 434 × 10^9^/L, and c-reactive protein (CRP)3.1 mg/L. Erythrocyte sedimentation rate fluctuated between 18–42 mm/hr. The blood and cerebrospinal cultures were negative. He was negative for influenza, parainfluenza, respiratory syncytial virus, syphilis, mycoplasma antibodies, EV71-IgM, TORCH, and autoantibodies. Humoral immune index: IgA 0.05 g/L, IgG 4.16 g/L, IgM 0.21 g/L. Cellular immune index: CD3+63%, CD4+40.6%, CD8+20.6%, CD4+/CD8+2.0, CD19 CD23 11.5%. Craniocerebral and abdominal ultrasound were unremarkable. B-mode ultrasonography of the neck revealed several enlarged lymph nodes on the left side, up to about15 × 7 mm. His chest X-ray was unremarkable. Echocardiography showed normal.

**Figure 1:**
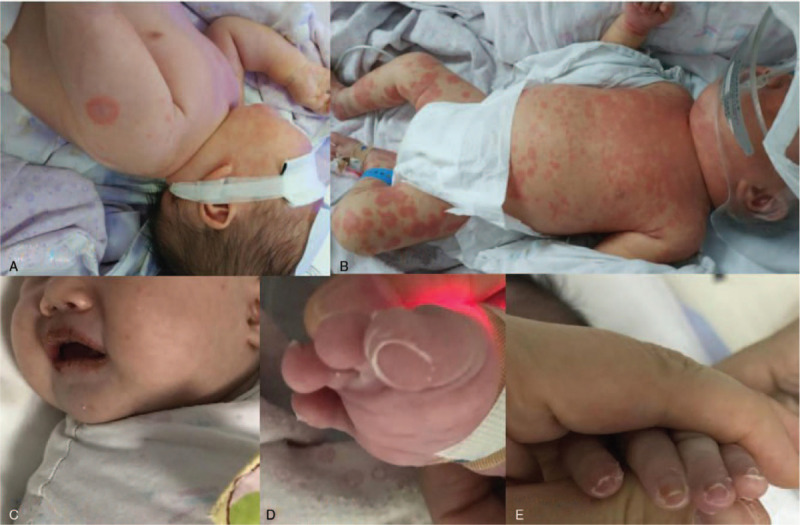
Clinical manifestations of our case.

Diagnosis and treatment: After admission, cephalosporin was given anti-infection and supportive treatment, and fever remained after 2 days treatment, with a peak of 39°C, and the rash gradually increased and faded under pressure, mainly in hands, feet, BCG erythema, shoulders and front chest (Fig. 1A/B). Polymorphous erythema was noted over the boy's entire body on the third day, and the CRP levers rose to 44.2 mg/L, procalcitonin rose to 0.63ng/ml, and the antibiotic was changed to cefoperazone sulbactam, and methylprednisolone 1 mg/kg was used. The rash and fever persisted, the antibiotic was changed to Meropenem, methylprednisolone was added to 2 mg/kg, and he was given intravenous immunoglobulin (IVIG) 800 mg/kg 1 time for supportive treatment. The temperature of the child gradually decreased (36.4–37.6°C), the rash subsided, but not completely disappeared, and cracked lips appeared on the 5th day (Fig. 1C). On the 7th day, the body temperature was higher than before, on the 9th day, appeared several enlarged lymph nodes on the left side, on the 10th day, appeared periungual desquamation, and the platelet count progressively increased from 434 × 10^9^/L (day 1) to 762 × 10^9^/L (day 10). The patient had classical features of complete Kawasaki disease (fever for 5 days or more with conjunctivitis, mucositis, extremity changes, rash with or without cervical lymphadenopathy ^[2]^). The patient was given IVIG (2 g/kg) and aspirin. Within 48 hour after the start of this therapy, the fever declined, the chapped lips got better, and the rash resolved, and distal extremity desquamation has appeared (Fig. 1D/E). On the 5th,13th 18th day, echocardiograms were normal with no carditis or coronary artery abnormalities. After 20 days of hospitalization, he was discharged to continue taking aspirin orally. The patient was followed for 3 months, and no abnormality was found in the blood routine and CRP. Follow-up echocardiography examinations showed normal on the 3rd and 12th month.

## 3 Discussion and conclusions

The clinical characteristics, laboratory examination, treatment and follow-up of 1 case of NKD in Children's Hospital of Soochow University in December 2018, were retrospectively analyzed. Our literature review encompassed a thorough search of the electronic databases of PubMed and Chinese databases for KD presenting in the neonatal period from January 2000 to August 2020. The key terms used in the search included newborns, neonates, Kawasaki Disease and Mucocutaneous lymph node syndrome. English and Chinese articles were reviewed. Clinical, laboratory, therapeutic and outcome data were collected.

Our literature search revealed only a few reported cases of suspected NKD worldwide (19 cases, including 15 English literature and 4 Chinese literature) (Table 1),^[5–20]^ together with this case, 20 cases in total. In all the presented cases including ours, 13 were male and 7 were female, the age of onset was 1 to 26 days. Six patients were complete KD (30%), 14 patients were incomplete Kawasaki disease (IKD). Table 2 summarizes the frequency of the clinical and laboratory manifestations of KD in our neonatal cases. Fever, rash, and extremity changes were reported in most infants, oral changes and thrombocytosis in close to two thirds. Among the 11 patients with coronary artery changes, 1 had an unknown prognosis due to loss of follow-up (patient 15). One patient died 2 days after discharge due to multiple organ failure (patient 17). 5 cases returned to normal (patient 3.6.7.14.20). The coronary artery changes in 4 cases still did not return to normal and were not followed up for a long time. Of the 20 NKD patients, 17 were sensitive to IVIG, 1did not use IVIG, and 1 was not sensitive to IVIG, and due to multiple organ failure, the parents gave up treatment and died 2 days after discharge. KD is an acute systemic vasculitis of childhood, and 76% of affected children are < 5 years of age, boys with the disease outnumber girls by about 1.5–1.7:1.^[21–23]^ Approximately 4% to 17% of patients are <6 months of age.^[24,25]^ The Japanese survey found that baby KD (including newborns) showed an atypical clinical manifestation.^[24]^ The incidence of KD varies greatly in the world. NKD is rare, and even in Japan with a high incidence of KD, NKD has not been reported much. According to Hangai et al^[5]^ between January 2001 and December 2012, only 23 of the 130243 KD children in Japan were newborns (1/5500). In 20 cases including ours, six patients had complete KD (30%),14 patients were IKD. Similar to Altarmmar et al^[7]^ (complete KD 25%) and Hangai et al.^[5]^ (complete KD 35%) reported. The proportion of KD in infants <12 months was 1/1500, IKD 15 (65%) in 23 cases, combined with coronary changes in 4 cases (17%). Coronary artery aneurysms or coronary dilation was present in 15% to 25% of children with KD,^[26]^ and these conditions might give rise to ischemic heart disease or sudden death.^[27–29]^ IVIG treatment could reduce the incidence of coronary artery abnormalitys to about 3% to 5%,^[2,26]^ but approximately 20% to 30% of KD patients had no response to IVIG.^[30,31]^ KD has seasonal epidemiological features. In Japan, a distinct peak of KD incidence was observed in January, as well as a more gradual increase in summer. This pattern supports the hypothesis that the disease is caused by multiple infectious agents, 1 prevalent in winter and another in summer.^[32]^ Genetic determinants have been suggested to contribute to KD susceptibility. Firstly, the risk of KD in siblings of affected children was ten times higher as compared with the general population.^[33]^ Secondly, it was twice as high in children born to parents with a history of KD compared with the general population.^[34]^ Thirdly, populations in Asian countries have higher incidence rates of KD than those in Western countries: Japan had the highest annual incidence rate,^[35]^ followed by Korea.^[36]^ In this article, we retrieved 5 cases of NKD in China, including our case, there were 6 cases in total, 5 were male and 1 was female, 2 patients had complete KD (33.3%), 4 patients were IKD. They all had fever, a total of 5 cases were late neonates (83.3%), 4 cases (66.6%) of peeling, 4 cases (66.6%) of skin rash, 4 cases (66.6%) of oral mucosal changes (lips red/cleft or strawberry tongue), 5 cases (83.3%) of coronary artery changes, 3 cases (50%) of conjunctival congestion, 3cases (50%) of cervical lymph node enlargement, 2 cases (40%) of limb edema. One patient died. 3 cases were normal (50%). The coronary artery changes in 1 case still did not return to normal when she was 3-year-old. The ratio of cervical lymph node enlargement and coronary artery dilation was significantly different from that of 20 cases, which was considered to be related to the small number of NKD in China.

**Table 1:** Literature review: summary of the case reports of neonatal kawasaki disease.

Pt	1	2	3	4	5	6	7	8	9	10	11	12	13	14	15	16	17	18	19	20
Ref	7	6	19	5	18	8	17	9	9	9	10	10	11	12	13	14	16	15	PR	20
Diagnosis	IKD	IKD	IKD	IKD	IKD	IKD	IKD	IKD	IKD	IKD	IKD	IKD	IKD	CKD	CKD	CKD	CKD	CKD	CKD	IKD
Age at onset/d	15	19	26	22	24	1	9	21	14	16	18	16	8	8	16	20	1	10	24	14
Sex	M	M	M	F	F	M	M	F	F	M	M	M	M	F	F	F	M	M	M	M
Fever duration (days)	5	0	10	4	12	0	19	4	3	4	6	9	>9	9	13	UN	>5	5	12	18
Rash	+	+	+	+	-	-	-	+	+	+	+	+	+	+	+	+	+	+	+	+
Oral changes^∗^	-	+	-	-	+	-	+	+	+	+	+	+	-	+	+	+	+	+	+	-
Extremity edema	+	+	+	-	-	-	-	+	+	+	+	+	-	+	+	+	+	+	-	-
Peeling	+^‡^	UN	+^‡^	+^‡^	-	-	+^‡^	UN	+^‡^	UN	+^‡^	UN	+^‡^	+	UN	+	+^‡^	+^‡^	+^‡^	+
Conjunctival congestion	+	+	-	-	+	-	-	-	-	-	-	-	-	+	+	-	+	+	-	+
Cervical lymph node enlargement	-	-	-	-	-	-	+	-	-	-	-	-	-	-	-	-	+	-	+	-
CAA^†^	-	-	LCA+	-	BCA+	BCA+	LCA+	-	-	-	-	-	+^§^	LCA+	+^§^	BCA+	BCA+	+^§^	-	+
CRP	N	H	H	H	H	UN	H	N	N	N	N	H	H	H	H	H	H	N	H	H
ESR	N	N	H	N	N	N	H	N	N	N	UN	UN	H	UN	H	N	UN	UN	H	H
Other	Apnea, ALTrise	Asphyxis, RDS	Anemia, Hypoalbuminema	-	meningitis	Anemia, jaundice	N	vomit	ALT rise	ALT rise, troponin rise	pneumonia	Cough, diarrhea	myocardial ischemia, hypertension	MR/TR/	MR/TR	MR/AR	Asphyxis, meconium inhaled	Apnea	pneumonia	bilateral axillary artery aneurysms
Platelet	H	N	H	N	H	dicline	H	UN	N	N	H	H	H	UN	H	H	N	H	H	H
IVIG response	+	+	+	+	+	Not use	+	+	+	+	+	+	+	+	+	+	-	+	+	+
CA outcome	N (1 year)	N (163 days)	N (3 years)	N (3 months)	AB (3 years)	N (1 year)	N (6 months)	N (6 weeks)	N (6 months)	N	N (6 weeks)	N (11 years)	AB (9 weeks)	N (6 weeks)	UN	AB (8 weeks)	Death	AB (2.5years)	N (1 year)	AB (7 months)

**Table 2:** Frequency of key clinical and laboratory features of 16 cases of neonatal Kawasaki disease.

Clinical and Laboratory Features	Frequency	(%)
Fever (any duration)	18/20	90
Duration of fever ≥ 5 days^∗^	14/19	73.6
Rash	17/20	85
Extremity changes	14/20	70
Oral changes	14/20	70
Conjunctival congestion	8/20	40
Cervical lymph node enlargement	3/20	15
Cardiac complications	11/20	55
Normal CRP^†^	6/19	31.5
Thrombocytosis^‡^	12/18	66.6
Elevated ESR^§^	6/15	40

Due to the lack of characteristic laboratory indicators, the diagnosis of KD is mainly dependent on clinical manifestations. Neonatal immune system development is not perfect, clinical symptoms are not typical with low incidence, so it is not easy to be noticed. Okazaki et a^[6]^ reported that 1 case of NKD without fever was a premature infant, with a rash as the first manifestation, accompanied by red lips, conjunctival congestion and acral edema, and IVIG was effective for treatment. In another case, coronary artery dilatation occurred on the first day after birth without other KD features, after excluding other diseases that could cause coronary artery dilatation, pericardial effusion, decreased PLT and hyperbilirubinemia,^[37]^ and IKD was diagnosed retrospectively. In addition to fever, the most common clinical manifestation was acral extremity change. Among the 20 cases, there were 13 cases of acral extremity edema in the early stage, and 5 cases of peeling after IVIG treatment without acral extremity edema in the stage (patient 4, 7, 13, 19, 20). Other clinical manifestations, in descending order of frequency, were skin rash, oral mucosal changes, conjunctival congestion, and cervical lymph node enlargement. Because oral mucosa changes and conjunctival congestion are transient, if there are any omissions in the medical history or physical examination, it is easy to misdiagnose. Neck lymph node enlargement accounts for only 15%, which is consistent with the literature conclusion of Manlhiot et al.^[38]^ CRP was normal in 6 (31.5%) of the 20 NKD cases (with 1 unreported CRP value). In our case, CRP was normal in the acute phase and began to rise after 4 days of illness. Elevated infection-related indicators are more common in older children, making the diagnosis of NKD particularly difficult in infants and young children.

The 20 cases of NKD were mostly late neonates, and only 2 (10%) of them were early neonates, which may be related to the immunity of the infant.

Echocardiography was performed in 20 cases with KD, and 11 of them had coronary changes (55%), which was much higher than that in older children, and also higher than Hangai et al (20%).^[5]^ If the children had coronary changes, KD was easier to be diagnosed with other signs and symptoms, while those without coronary changes were more likely to be missed.

The treatment for KD is the same as that for IKD, the preferred scheme is the combination of immunoglobulin and aspirin, which can rapidly reduce fever and improve clinical symptoms while reducing the incidence of coronary artery disease. But approximately 20% to 30% of KD patients had no response to IVIG.^[30,31]^ Currently, prednisolone (PSL) is used for the treatment of IVIG-resistant KD. A pilot study (RAISE study) showed the efficacy of first dose IVIG plus PSL in reducing inflammation in severe KD, compared with IVIG alone.^[39]^ The infliximab may be considered as an alternative to a second infusion of IVIG or corticosteroids for IVIG-resistant patients. The cyclosporine may be considered in patients with refractory KD in whom a second IVIG infusion, infliximab, or a course of steroids has failed. The immunomodulatory monoclonal antibody therapy, cytotoxic agents, or plasma exchange may be considered in highly refractory patients who have failed to respond to a second infusion of IVIG, an extended course of steroids, or infliximab.^[2]^

In conclusion, neonatal febrile diseases are easily misdiagnosed as sepsis, viral infection or immune diseases. If NKD loses the best time for IVIG treatment, aggravating the body's immune response and eventually developing into coronary artery damage. It is essential to maintain a high index of suspicion for NKD in febrile neonates which fail to respond to antibiotics, and attention should be paid to the observation of skin rash, changes in oral and lip mucosa, changes in acral extremity edema, and timely complete the Echocardiography examination, early detection, early intervention may appear coronary artery damage and long-term damage. The low incidence of NKD may be related to passive immunity from maternal antibodies.^[40]^ Besides, NKD is more manifested as an incomplete type, which may be related to the immature immunity of neonates.

## 4 Strengths and limitations

(1) The clinical characteristics, laboratory examination, treatment and follow-up of 1 case of NKD were retrospectively analyzed.
(2) Our literature review encompassed a thorough search of the electronic databases of PubMed and Chinese databases for KD presenting in the neonatal period from January 2000 to August 2020.
(3) A detailed qualitative approach was used, English and Chinese articles were reviewed.
(4) the shortcoming of this paper is that the number of cases studied is too small, and we need more case studies to be more convincing.

## Author contributions

CL, YD participated in the study design and writing of the manuscript. HW participated in clinical data collection. GW carried out the interpretation of data. XZ participated in data analysis, interpretation of data and writing of the manuscript. All authors read and approved the final manuscript.

**Data curation:** Huawei Wang.

**Formal analysis:** Gaohong Wu.

**Writing – original draft:** Cancan Li, Yiming Du.

**Writing – review & editing:** Xueping Zhu.
